# A clinical perspective on the utility of alpha 1 antichymotrypsin for the early diagnosis of calcific aortic stenosis

**DOI:** 10.1186/s12014-017-9147-z

**Published:** 2017-04-21

**Authors:** Tatiana Martin-Rojas, Laura Mourino-Alvarez, Felix Gil-Dones, Fernando de la Cuesta, Esther Rosello-Lleti, Carlos M. Laborde, Miguel Rivera, Luis Fernando Lopez-Almodovar, Juan Antonio Lopez, Finn Akerstrom, Luis R. Padial, Maria G. Barderas

**Affiliations:** 1 Department of Vascular Physiopathology, Hospital Nacional de Parapléjicos, SESCAM, Edificio de Terapia 2ª Planta, Toledo, 45071 Toledo Spain; 2 Cardiocirculatory Unit, Health Research Institute, Hospital La Fe, Valencia, Spain; 3 Cardiac Surgery, Hospital Virgen de la Salud, SESCAM, Toledo, Spain; 4 Unidad de Proteomica CNIC, Madrid, Spain; 5 Department of Cardiology, Hospital Virgen de la Salud, SESCAM, Toledo, Spain

**Keywords:** Calcific aortic stenosis, Multi-proteomic, Alpha 1 antichymotrypsin, Biomarker

## Abstract

**Background:**

Calcific aortic stenosis (CAS) is the most common heart valve disease in the elderly, representing an important economic and social burden in developed countries. Currently, there is no way to predict either the onset or progression of CAS, emphasizing the need to identify useful biomarkers for this condition.

**Methods:**

We performed a multi-proteomic analysis on different kinds of samples from CAS patients and healthy donors: tissue, secretome and plasma. The results were validated in an independent cohort of subjects by immunohistochemistry, western blotting and selected reaction monitoring.

**Results:**

Alpha 1 antichymotrypsin (AACT) abundance was altered in the CAS samples, as confirmed in the validation phase. The significant changes observed in the amounts of this protein strongly suggest that it could be involved in the molecular mechanisms underlying CAS. In addition, our results suggest there is enhanced release of AACT into the extracellular fluids when the disease commences.

**Conclusions:**

The significant increase of AACT in CAS patients suggests it fulfils an important role in the physiopathology of this disease. These results permit us to propose that AACT may serve as a potential marker for the diagnosis of CAS, with considerable clinical value.

## Background

Calcific aortic valve stenosis (CAS) is the most prevalent heart valve disease in Europe and North America, and it is currently responsible for most of the aortic valve replacement procedures that are performed [1, 2]. CAS is age-related and given the aging population in occidental countries, its prevalence is expected to increase in the forthcoming decades [3]. The natural history of CAS involves a long clinically silent phase of valve calcification and hardening (valve sclerosis) that generally requires at least a decade before heralding the clinical disease. CAS is characterized by an initial stage that has a pathogenesis similar to atherosclerosis, and an active pathology that is characterized by lipid accumulation, inflammation and calcification [4]. Hemodynamically, CAS involves obstructed blood flow at the level of the aortic valve, with a pressure gradient between the left ventricular (LV) and the ascending aorta. The increased LV pressure enhances myocardial wall stress, which is at first compensated by LV hypertrophy, but that is followed by LV dilation and systolic dysfunction during the later stages of the disease [5].

At present, aortic valve replacement is the only effective treatment for severe symptomatic CAS, whereas in most asymptomatic patients the risk of surgery outweighs that of disease monitoring. Therefore, asymptomatic patients with CAS are managed through regular clinical and echocardiography check-ups, as well as basic education to modify their lifestyle and to provide awareness of cardiac risk factors [6]. Nevertheless, it is necessary to develop markers that can guide clinicians to classify patients at high or low risk of disease progression and subsequently tailor their follow-up frequency, thereby optimizing healthcare resources and reducing costs [7]. The possibility of predicting the development of CAS in patients would be tremendously useful.

**Fig. 1:**
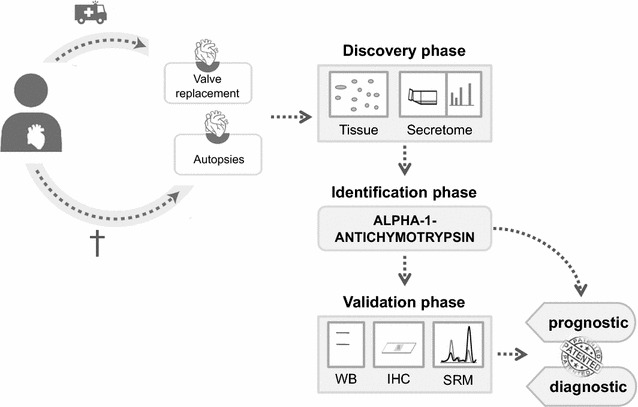
Schematic representation of the workflow. Samples were collected from valve replacement surgeries (AS) and autopsies (controls). During the discovery phase the whole tissue and secretome were analyzed and AACT was identified. Finally, AACT was validated as a potential biomarker in WBs, and by IHC and SRM

Here, we have adopted an omics strategy to perform an unbiased, non-targeted study of CAS. Our data show the importance of the alpha 1 antichymotrypsin (AACT) protein in CAS, for the first time describing its potential as a future diagnostic marker of this disease. This protein is associated with the acute-phase of the disease, placing the focus on inflammatory processes in relation to the mechanisms underlying CAS. Moreover, the potential predictive value of AACT was validated through a selected reaction monitoring (SRM) analysis in plasma samples, which could facilitate its future implementation in clinical practice (Fig. 1).

## Methods

### Patient and control samples

Stenotic heart valves (n = 20) were obtained from severe symptomatic CAS patients (55% male, 45% female), with an average age of 74 (SD = 4.0) years, who underwent aortic valve replacement. All patients had hypertension, 50% suffered hyperlipidemia and 60% diabetes mellitus. Patients with a medical history of significant aortic regurgitation, rheumatic fever or morphological valve alterations consistent with rheumatic heart disease, we’re excluded from the study (Table 1). The valves were classified according to a macroscopic inspection performed blind by cardiovascular surgeons. Normal valves (n = 20) from the autopsies of subjects who had died due to non-cardiovascular diseases were obtained within 4 h of death and used as controls (Table 2). In all the cases, the structure of both the stenotic and non-stenotic valves was 3-cuspid.

**Table 1:** Clinical characteristics of AS patients

Patient number	Age/gender	AHT	Diabetes	Dyslipidemia
1	74/F	Yes	No	Yes
2	73/M	Yes	Yes	No
3	68/M	Yes	No	Yes
4	81/M	Yes	No	Yes
5	69/M	Yes	Yes	Yes
6	79/F	Yes	Yes	No
7	73/F	Yes	Yes	Yes
8	77/M	Yes	No	No
9	79/F	Yes	Yes	No
10	75/M	Yes	No	Yes
11	74/M	Yes	Yes	Yes
12	74/F	Yes	Yes	No
13	79/F	Yes	Yes	No
14	79/F	Yes	Yes	No
15	72/F	Yes	No	No
16	63/M	Yes	No	Yes
17	72//M	Yes	No	Yes
18	75/F	Yes	Yes	Yes
19	78/M	Yes	Yes	No
20	74/M	Yes	Yes	No
Mean	74 ± 0/45% F–55% M	100.00%	60.00%	50.00%

*F* female, *M* male, *AHT* arterial hypertension

**Table 2:** Clinical characteristics of control subjects

Control number	Age/gender	Cause of the death	AHT	Diabetes	Dyslipidemia
1	64/M	Bronchopneumonia	No	No	No
2	51/F	Septic shock	Yes	No	No
3	47/H	Sepsis	No	No	No
4	47/F	Bacterial pneumonia	No	No	No
5	47/F	Carcinogenesis	No	No	No
6	34/M	Acute Pancreatitis	Yes	No	No
7	64/F	Bacterial Sepsis	No	No	No
8	78/F	Bronchopneumonia	No	No	No
9	42/F	Lymphoma	No	No	No
10	67/M	Renal insufficiency	No	No	No
11	74/M	COPD	No	No	No
12	87/F	Carcinoma	No	No	No
13	48/F	Pulmonary thromboembolism	No	No	No
14	55/M	Pneumonia	No	No	No
15	87/F	Cholelithiasis	No	No	No
16	76/M	Carcinoma	No	No	No
17	80/F	Atrial fibrillation	Yes	Yes	No
18	49/F	Respiratory insufficiency	No	No	No
19	74/M	Septic shock	Yes	No	Yes
20	77/F	COPD	Yes	No	No
Mean	69 ± 7.07/40% M–60% F		25.00%	5%	5%

*F* female, *M* male, *AHT* arterial hypertension, *COPD* chronic obstructive pulmonary disease

For the SRM validation analysis, peripheral blood samples were collected from CAS patients (n = 8) and control subjects (n = 8) selected to avoid significant differences between the groups in terms of age (*p* = 0.6), gender (*p* = 0.32), obesity (*p* = 0.5), hypertension (*p* = 0.5), dyslipidemia (*p* = 0.5) and diabetes (*p* = 0.7: Table 3). This study was carried out in accordance with the recommendations of the Helsinki Declaration and it was approved by the ethics committee at the Hospital “Virgen de la Salud” (Toledo, Spain). Signed informed consent was obtained from all the subjects or their relatives (in case of necropsies) prior to their inclusion in the study.

**Table 3:** Clinical characteristics of the subjects used in the validation phase

Subject number	Age/gender	AHT	Diabetes	Dyslipidemia
1 Control	62/M	Yes	No	Yes
2 Control	82/M	Yes	No	No
3 Control	86/M	Yes	No	Yes
4 Control	96/M	No	Yes	Yes
5 Control	78/F	Yes	Yes	No
6 Control	81/M	Yes	No	No
7 Control	40/F	No	No	No
8 Control	30/M	No	No	No
9 AS	77/F	Yes	Yes	No
10 AS	86/M	Yes	No	No
11 AS	69/F	Yes	No	No
12 AS	77/M	No	No	No
13 AS	60/F	Yes	No	No
14 AS	75/F	Yes	No	Yes
15 AS	74/M	No	No	Yes
16 AS	74/F	Yes	Yes	No
Mean control	75% M	62.75%	25%	37.75%
Mean AS	37.5% M	75	25%	25%

*F* female, *M* male, *AHT* arterial hypertension

### Tissue sample preparation

Aortic valves were preserved and transported in PBS, and processed within a maximum of 2 h after extraction. The valves were washed immediately in PBS to reduce blood contaminants. For 2D-DIGE and western blotting, one aortic valve leaflet was ground into a powder in liquid N_2_ in a mortar. Protein extracts were then prepared from the valve as described previously [8, 9] and the total protein concentration was measured by the Bradford-Lowry method (Bio-Rad protein assay) [10].

For secretome analysis, the second valve leaaflet was cultured as described elsewhere [11, 12] using medium supplemented with antibiotics and amphotericin B (Fungizone^®^) to avoid contamination. Samples were transferred to a Petri dish (Cell Star^®^), cut into pieces and incubated at 37 °C in lysine-arginine free 1640 RPMI medium (Cell Culture Technologies Invitrus) supplemented with 5 mg/ml fungizone, 250 mg/ml amikacin, 2 mg/ml l-lysine 2HCl (U-13C6, 97–99%) and 10 mg/ml l-Arginine HCl (U-13C6, 97–98%: Cambridge Isotope Laboratories Inc., Andover, MA) in a humidified atmosphere of 5% CO_2_. The valves were cultured for 96 h and the medium collected was stored at −80 °C until analysis. Finally, the valves were fixed in formalin at 4 °C, decalcified in Shandon-TBD1 (Thermo Scientific) and embedded in OCT for subsequent immunohistochemistry.

### Plasma sample preparation

Blood samples were collected in tubes containing EDTA and centrifuged at 1125×*g* for 15 min. The resulting supernatant was frozen immediately at −80 °C until analysis.

### 2D-DIGE separation, image acquisition and analysis

Tissue proteins were labeled according to the manufacturer´s instructions (GE Healthcare), and as described by Gil-Dones and Martin-Rojas [8, 9]. In all experiments an internal standard was added containing equal amounts of each protein extract. The internal standard and protein extracts from one stenotic and one control valve were combined and run on a single gel (150 μg of total protein). The proteins were separated by isoelectric focusing in the first dimension and the strips were then equilibrated via two consecutive incubations in SDS-equilibration buffer (1.5 M Tris∙HCl [pH 8.8], 6 M Urea, 87% Glycerol and 2% SDS) plus dithiothreitol and iodoacetamide, respectively. Subsequently, the proteins were separated on 12% Acrylamide/Bisacrylamide gels using an EttanDalt Six device (GE Healthcare), which were then scanned using a Typhoon 9400 fluorescence gel scanner (GE Healthcare). Relative protein quantification of Aortic Stenosic (AS) and healthy valves was performed with DeCyder software v6.5 (GE Healthcare) and with the multivariate statistical module EDA (Extended data analysis). Only proteins with >1.5-fold differences in abundance were considered significant. A statistical analysis was then carried out to determine the changes in protein expression, with *p* values below 0.05 accepted as significant when the Student’s *t* test was applied.

### Protein identification by MALDI-TOF/TOF

2D-DIGE gels were silver stained to visualize the protein spots, and all differentially expressed protein spots were then manually excised and identified at the Hospital Nacional de Paraplejicos’ Proteomics Unit. The proteins were automatically digested in an “Ettan Digester” (GE Healthcare) according to Shevchenko et al. [13], with minor modifications. An aliquot of each digestion was mixed with an aliquot of the matrix solution and this mixture was pipetted directly onto the stainless steel sample plate of the mass spectrometer.

The MALDI-MS/MS data were obtained in an automated analysis loop using a 4800 Plus MALDI TOF/TOF Analyzer (Applied Biosystems). The spectra were acquired and the mass data were analysed automatically with the 4000 Series Explorer Software version 3.5.3 (Applied Biosystems). MALDI-MS and MS/MS data were combined through the GPS Explorer Software (Version 3.6) to search a non-redundant protein database (Swissprot 56.5) using the Mascot software (version 2.2: Matrix Science) [14].

### LC–MS/MS identification

Labeled peptide samples from secretome experiments were analyzed by LC–MS/MS using a C-18 reversed phase nano-column (75 µm I.D. × 50 cm, 2 µm particle size, nanoEASY 100 C18: Thermo Fisher Scientific) and a continuous acetonitrile gradient of: 0–30% B in 420 min, 30–43% A in 5 min, and 43–90% B in 1 min (where: A = 0.5% formic acid; B = 90% acetonitrile, 0.5% formic acid). A flow rate of 200 nL/min was used to elute the peptides from the reverse phase nano-column to an emitter nanospray needle for real time ionization and peptide fragmentation in a QExactive Hybrid Quadrupole-Orbitrap mass spectrometer (Thermo Fisher Scientific).

### Western blotting

Protein extracts from CAS and control valves were resolved by 12% SDS-PAGE using a Bio-Rad Miniprotean II electrophoresis unit run for 1 h at a constant current of 25 mA/gel. After SDS-PAGE, the proteins were transferred to a nitrocellulose membrane under a constant voltage of 15 V for 20 min. Ponceau S staining was performed to guarantee that equal amounts of aortic valve proteins were loaded onto the IPG strips (2-D western blotting) or onto polyacrylamide gels (regular western blotting). Subsequently, the membranes were blocked for 1 h and incubated overnight with the primary antibody at a concentration of 1 µg/ml (Abcam, Ref. ab9374) in PBS-Tween20 + 5% non-fat dry milk. After rinsing, the membranes were incubated with the specific HRP-conjugated secondary antibody in PBS-Tween20 + 5% non-fat dry milk, which were detected by enhanced chemiluminescence (ECL: GE Healthcare) following the manufacturers’ instructions. Protein band intensity was measured on a GS-800 Calibrated Densitometer (Bio-Rad).

### Immunohistochemistry

Tissues were embedded in OCT and cryosections (6 µm) were obtained for immunohistochemical analyses. The sections were blocked with 10% BSA in PBS with 0.1% Tween 20 and incubated overnight at 4 °C with the primary antibody (10 µg/ml: Abcam, ab9374). The sections were then incubated with an IgG-HRP-conjugated secondary antibody (NORDIC Immunology) and the chromogenic reaction was developed using 3,3′-diaminobenzidine. Sections were counterstained with hematoxylin prior to dehydration and coverslipping. As a negative control, the complete immunohistochemical procedure was performed on adjacent sections without adding the primary antibody. To quantify the DAB stained area, an orthonormal transformation of the RGB images was applied using an ImageJ plugin based on Ruifrok and Johnston’s method for color deconvolution.

### Selected reaction monitoring

Prior to mass spectrometry (MS) analysis in the LC–MS/MS system, crude plasma samples were reduced, digested and cleaned on Pep-Clean spin columns (Pierce) according to the manufacturer’s instructions. LC–MS/MS was performed on a TEMPO nano LC system (Applied Biosystems) combined with a nano LC Autosampler coupled to a modified triple quadrupole (4000 QTRAP LC/MS/MS, Applied Biosystems). Theoretical SRM transitions were designed using MRMpilot software v1.1 (ABSciex), and the MIDAS acquisition method and Skyline were used for the analysis, including the theoretical transitions.

### Statistical analysis

Statistical analyses were performed using SPSS 15.0 software for Windows (SPSS Inc.). A Kolmogorov–Smirnov test was applied to evaluate the normal distribution of the continuous variables in the population analyzed. When a normal distribution was demonstrated, a comparison of the means was performed using a *t*-Student test. For populations with no normal distribution, the non-parametric Mann–Whitney U test was used. Discrete variables, such as sex or the presence/absence of risk factors, were compared using Fisher’s exact test. For all tests, statistical significance was accepted when *p* < 0.05.

## Results

**Fig. 2:**
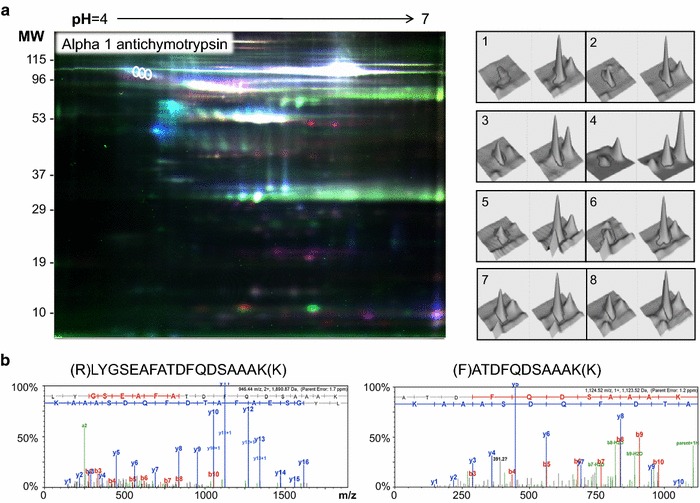
2D-DIGE results. **a** Representative fluorescence DIGE image, showing the differentially expressed spots corresponding to AACT. On the *right*, the spot intensity in 3D of one of the three spots corresponding to AACT is shown. The differences between controls and patients were consistent in the 8 gels studied. **b** Fragmentation spectra of the proteotypic peptides used for AACT identification by MALDI-MS/MS. The fragments of the *y* ion series are shown in *blue*, while the *b* ion series are shown in *red*

To identify proteins of interest in CAS, two complementary proteomic approaches were applied to two different types of CAS samples (tissue and secretome), the in-gel separation of proteins 2D-DIGE MALDI-MS/MS and liquid-chromatography separation of peptides (LC–MS/MS). The results obtained were validated in an independent cohort of plasma samples to support their translation to a clinical setting. In tissue 2D-DIGE analysis, we identified 3 spots in the CAS tissue that corresponded to AACT (Fig. 2). In all cases, the MASCOT score of these spots was higher than 70 and they were about twofold more intense in stenotic valves (Table 4).

**Table 4:** List of the alpha 1 antichymotrypsin isoforms identified in the 2D-DIGE analysis of AS patients versus controls

Protein name	Accesion number	MASCOT score	Seq coverage	Match peptides	Expect score	Ratio CAS/C	Theo. pI	Theo. MW	Exp. pI	Exp. MW
Alpha-1-antichymotrypsin	P01011	394	23	13	1.6e−034	1.96	5.33	47.62	4.7	95
		174	37	16	1.6e−012	1.94	5.33	47.62	4.65	104
		77	23	7	0.0074	2.00	5.33	47.62	4.6	105

The Uniprot accession number is shown, along with the MASCOT score, sequence coverage, number of matched peptides, expect score, variation between the patients and the control groups (ratio AS/C), theoretical isoelectric point (pI) and molecular weight (MW), and experimental isoelectric point (pI) and molecular weight (MW) in kDa

We also performed a secretome analysis using l-lysine 2HCl (U-^13^C_6_, 97–99%) and 10 mg/ml **l**-Arginine HCl (U-^13^C_6_, 97–98%). AACT was one of the secreted proteins that incorporated the labeled amino acids in the AS group alone. AACT was analyzed using the SecretomeP software [15] and it was predicted to be a secreted protein. Its signal peptide indicated secretion by the classical pathway and in the absence of the signal peptide, it had an NN-score >0.5, indicative of non-classical secreted proteins. As such, it seems likely that AACT is secreted by the classical pathway.

**Fig. 3:**
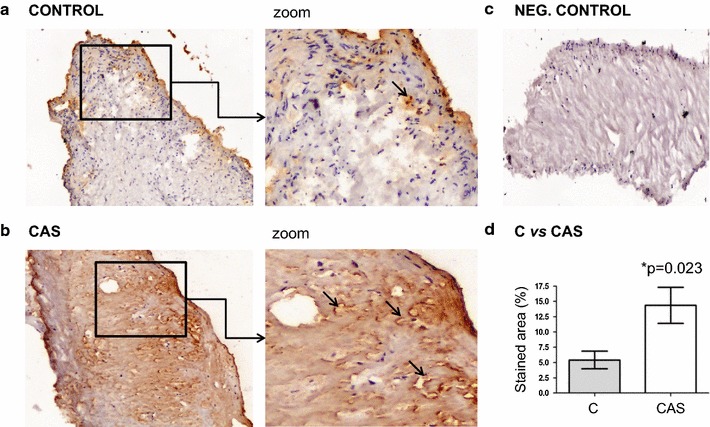
Validation of the AACT protein using IHC. Stronger expression of AACT is observed in the endothelium on the aortic side and, to a greater extent in the fibrosa of the stenotic valves when compared to the controls. DAB staining is brown and is indicated by the *arrows*. Statistical analyses showed significant differences (*p = 0.023). Amplified images at ×100 magnification

**Fig. 4:**
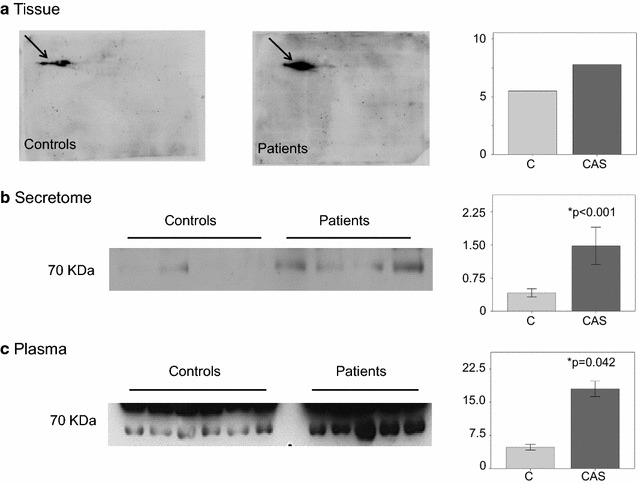
Validation of the AACT protein in Western Blots. **a** Bidimensional immunodetection analysis of tissue samples in which differences in the expression of the isoforms is indicated by *arrows*. **b** Immunodetection of the secretome samples. The band was more intense in the patient group than in the controls. **c** Immunodetection of the plasma samples. Again, the band in the plasma from the patients was more intense than in the controls. Quantification by densitometry is also shown in the figure and the AACT levels are clearly higher in the CAS patients in all cases

We validated these results in an independent cohort of control and CAS subjects by immunohistochemistry, in western blots and by SRM. AACT accumulated most strongly in the tissue from the CAS patients (Fig. 3), primarily in the endothelium on the aortic side and in the fibrosa (controls mean = 5.41 ± 1.43; CAS mean = 14.35 ± 2.951: *p* = 0.023). As indicated, the proteomic results were also validated in 2-D western blots using three different CAS samples: tissue, plasma and secretome (Fig. 4). The relative intensity of this protein was 1.5 higher in CAS tissue than in the control samples and we also found a significant increase in the plasma from CAS patients (mean = 18.00 ± 1.75) relative to the controls (mean = 4.82 ± 0.63: *p* = 0.042). Similar results were obtained when analyzing the secretome (controls mean = 0.41 ± 0.01; CAS mean = 1.48 ± 0.42: *p* < 0.001).

**Fig. 5:**
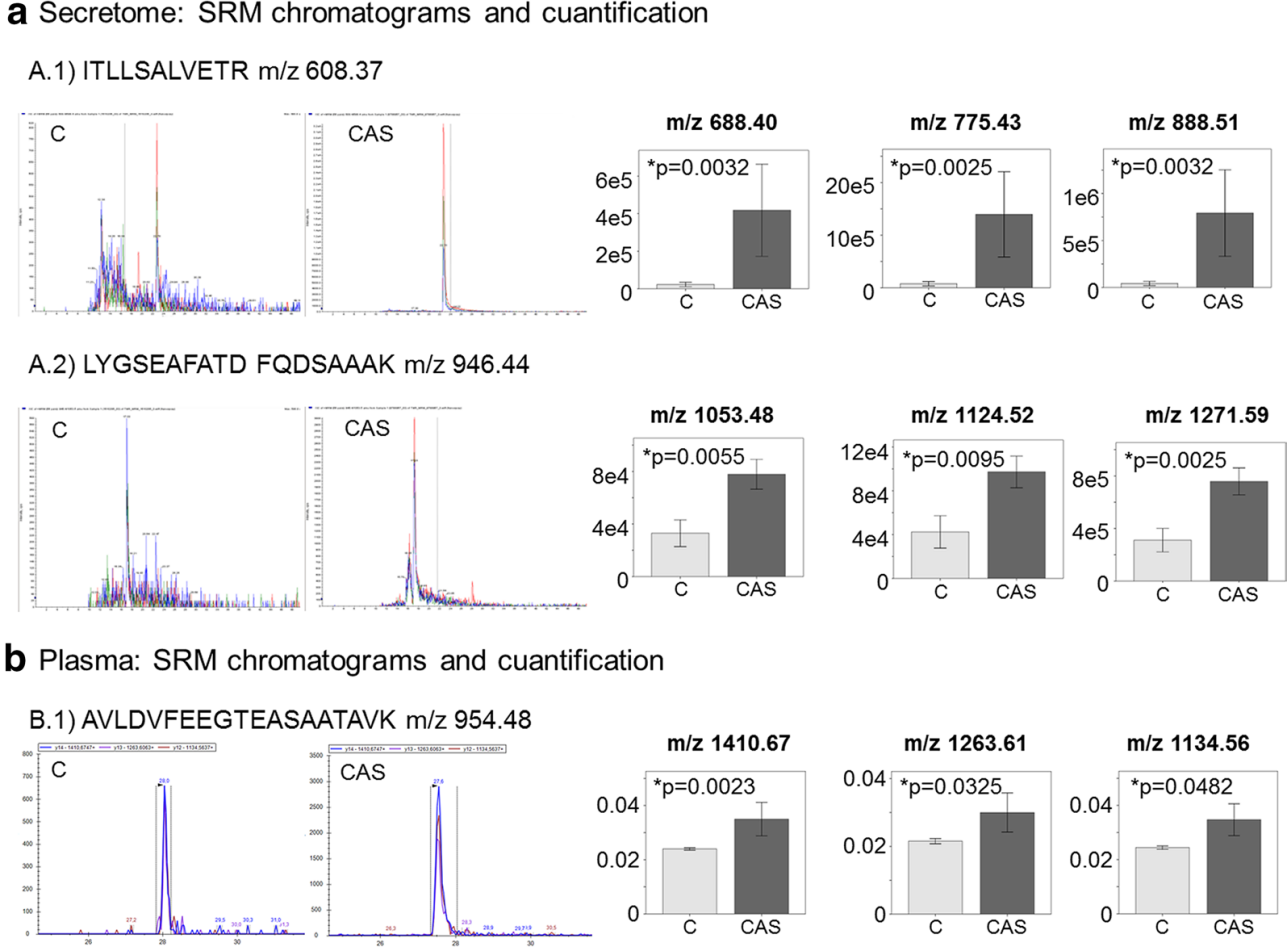
SRM validation of the differences in AACT found using LC-MS/MS. **a** Chromatograms of secretome samples showing the transitions of the 2 proteotypic peptides: *ITLLSALVETR* (*A.1*) and *LYGSEAFATDFQDSAAAK* (*A.2*). As it is shown in the figure, the m/z values selected in the *first* quadrupole were 608.37 and 946.44, respectively. On the *right*, quantification of the three transitions (*area under the curve*) that were monitored for each peptide. The different m/z values selected in the *third* quadrupole are indicated in the figure. **b** Chromatograms of plasma samples showing the transitions of the proteotypic peptide *AVLDVFEEGTEASAATAVK* (m/z 954.48) and quantification of each transition (*area under the curve*) in plasma samples. The different m/z values selected in the *third* quadrupole are indicated in the figure

In terms of the validation of the SRM secretome samples, two different peptides and three transitions of each of these were measured (Fig. 5). The expression of both was significantly higher in the CAS patients than in the controls, and significant differences between the two study groups were also evident in the plasma samples (Table 5).

**Table 5:** Statistical analysis of the SRM

	Peptide	T	Mean ± SEM	*p* value
1	ITLLSALVETR	T1	Control: 22,938 ± 11,790; CAS: 418,327 ± 245,046	0.0032
		T2	Control: 77,287 ± 41,645; CAS: 1,395,651 ± 811,858	0.0025
		T3	Control: 42,469 ± 22,863; CAS: 792,453 ± 460,476	0.0032
2	LYGSEAFATDFQDSAAAK	T1	Control: 32,992 ± 10,156; CAS: 77,827 ± 11,396	0.0055
		T2	Control: 42,505 ± 14,734; CAS: 97,228 ± 14,444	0.0095
		T3	Control: 31,132 ± 8909; CAS: 75,803 ± 10,252	0.0025
3	AVLDVFEEGTEASAATAVK	T1	Control: 0.024 ± 0.001; CAS: 0.035 ± 0.006	0.0023
		T2	Control: 0.022 ± 0.001; CAS: 0.030 ± 0.006	0.0325
		T3	Control: 0.024 ± 0.001; CAS: 0.035 ± 0.006	0.0482

The peptide sequence is shown, along with the selected transitions (T) and the statistical significance (*p* value)

## Discussion

Aortic stenosis is a progressive disease with aortic valve sclerosis as the first manifestation, which ultimately leads to valve dysfunction [4]. Over the last few years, great efforts have been made to understand the physiological and pathological mechanisms implicated in CAS. Calcification of the native aortic cups in this disease is a complex process, which appears to be related to inflammation and ossification [16]. Nevertheless, the mechanisms underlying degenerative heart valve disease still remains largely unclear, especially when compared with our understanding of the mechanisms underlying other cardiovascular diseases like heart failure and atherosclerotic disease. Due to the high social costs associated with CAS healthcare, there is a strong unmet clinical need to find markers that aid early diagnosis, estimate the long-term prognosis, monitor treatment response and predict potential adverse effects in CAS patients. By using multi-omics approaches, we hoped to define markers that may potentially serve to diagnose the disease earlier than traditional methods, such as echocardiography [11]. Novel tools for easier diagnosis will reduce the high morbidity and mortality associated to surgery, and they may provide a solution for those patients who cannot be operated on, as well as improving patient management.

The use of different proteomic techniques on distinct samples allowed us to identify AACT as a potential diagnostic marker for CAS. This was validated in an independent cohort of subjects using orthogonal techniques to analyze tissue, the secretome and plasma samples. Taking into account these results, AACT detection and its quantification could define AS, and as we believe that AACT may be a potential diagnostic marker of CAS, which have assessed its patentability (IP2145.5).

Although the exact role of AACT in CAS is unknown, it has been postulated to have several effects on the cardiovascular system. AACT is a serine protease inhibitor affecting acute phase proteins, and it induces tumor necrosis factor (TNF)-α and NF-κB expression [17], which both have anti-inflammatory properties. Additionally, AACT is thought to afford protection during ischemia reperfusion by inhibiting neutrophil accumulation into the ischemic-reperfused myocardium and by inactivating cytotoxic metabolites released from neutrophils [18]. This protein is a member of the serpin family [19] and it is also known as serpin 3. Although it is mainly synthesized in the liver [19–23], we have also found it expressed in aortic valves. It is well known that the levels of acute-phase proteins in plasma are elevated in response to inflammation and rupture [24, 25]. Thus, endothelial damage could explain the over expression of AACT in CAS, as well as the inflammation, which seems to be involved in the development of the disease [26–29].

Protein changes observed in tissue are important since they reveal the specific molecular processes taking place in the damaged organ. Nevertheless, valve tissue is not very accessible to clinicians and hence, it is significant that the increase in AACT is also evident in the secretome of CAS samples, as well as in the plasma of patients. The analysis of the secretome and plasma showed that this protein is released directly from the stenotic valve in response to the development of CAS, such that higher levels of ACCT imply further CAS development, which implies vascular surgery.

While, potential biomarkers found in plasma are less specific than those found in tissues, we have demonstrated the valvular origin of the increase in AACT in this biological fluid. This sample is easily accessible to clinicians, as extraction requires non-invasive methods and it is routinely used in clinical analysis. Besides, current methods such as echocardiography are time-consuming and expensive when compared with plasma analysis. Moreover, high qualified staff are needed to interpret images due to their complexity and thus it would be easier to measure AACT in a clinical setting in order to diagnose CAS. It is now important to perform a study with a larger number of patients with different grades of injury in order to confirm that AACT levels increase with the severity of the disease as a physiological response to the processes that take place in the stenotic aortic valve. This would allow us to establish ranges of protein concentrations in an attempt to predict which patients may develop a severe form of CAS, making it possible to apply more personalized treatment. In addition a prospective study would allow us to confirm the prognostic value of this protein.

## Conclusions

Alpha-1-antichymotrypsin seems to be an important indicator for clinicians regarding the progression of CAS. These data are important considering the aging of the population where cardiovascular diseases, in this case CAS, are becoming an increasing economic and social burden in Western countries. Future studies with plasma samples taken at different time points and taken from patients with distinct disease progression will be necessary to analyze the potential role of AACT as a prognostic and diagnostic marker of CAS in more depth.
